# Tertiary lymphoid structure topotypes in intrahepatic cholangiocarcinoma: spatial immunity, checkpoint-response associations, and therapeutic reprogramming

**DOI:** 10.3389/fimmu.2026.1919543

**Published:** 2026-08-20

**Authors:** Zhigao Yuan, Xingfei Li, Qian Cheng, Jie Gao

**Affiliations:** 1 Department of General Surgery, Civil Aviation General Hospital, Beijing, China; 2 Department of Hepatobiliary Surgery, Peking University People’s Hospital, Beijing, China; 3 Peking University Research Center for Diagnosis & Treatment of Liver Cancer, Beijing, China; 4 Peking University Institute of Organ Transplantation, Beijing, China

**Keywords:** immunotherapy, intrahepatic cholangiocarcinoma, radiomics, spatial immunology, stem-like CD8 T cells, tertiary lymphoid structures, translational medicine, tumor immune microenvironment

## Abstract

Tertiary lymphoid structures (TLS) have evolved from descriptive histological findings into candidate biomarkers and targetable immune niches in solid tumors. In intrahepatic cholangiocarcinoma (ICC), however, similar lymphoid architectures may have different biological implications depending on their relationship to tumor nests, immune circuitry, and suppressive stroma. We define a “TLS topotype” as the multidimensional state of an individual TLS-centered region, including the TLS core, its perilesional neighborhood, and the nearest tumor–stroma interface. This state integrates anatomic topology and tumor accessibility, structural maturity, putative effector circuitry, and suppressive context; treatment-induced plasticity is considered separately through longitudinal assessment. Because heterogeneous TLS may coexist within one tumor, the whole tumor is represented by a “topotype landscape” based on compartment-specific TLS density and the distribution of TLS-level states rather than a single label. Direct human ICC evidence supports compartment-resolved associations between TLS distribution and outcome and identifies suppressive B-cell, Treg, myeloid, and stromal programs. These observations establish spatial TLS organization as prognostically relevant, but do not validate the framework or demonstrate treatment-specific prediction. By contrast, TLS-resident TCF7-positive stem-like CD8 T-cell maintenance, coordinated B-cell–dendritic-cell–T-cell circuitry, and ICI-specific predictive value remain unproven in ICC and should be considered hypothesis-generating. The framework therefore distinguishes candidate circuit-competent, tumor-accessible TLS states from stromally excluded or suppressive-neighborhood states while allowing mixed profiles. Multiplex pathology and spatial transcriptomics may define these states, whereas radiomics, liquid biopsy, and pretreatment biopsy provide complementary but incomplete information. Biopsy can characterize only TLS states captured in sampled tissue, and radiomics currently estimates whole-lesion TLS probability rather than functional topology. Marker expression or spatial colocalization alone does not establish functional productivity, which requires evidence of tumor-antigen presentation or specificity, local lymphocyte renewal or clonal expansion, functional perturbation, or a spatially linked treatment response. By separating TLS-level state, tumor-level landscape, and patient-level trial stratum, this review provides a framework for biomarker development, therapeutic reprogramming, prospective validation, and longitudinal monitoring in ICC. No TLS topotype has yet been validated to predict differential benefit from immune checkpoint inhibition in ICC.

## Highlights

- The central innovation is to replace a panoramic TLS survey with a disease-specific translational framework centered on TLS functional topology.
- Intratumoral and peritumoral TLS should be interpreted as different immune ecosystems rather than as different positions of the same biomarker.
- A clinically useful TLS biomarker in ICC should integrate location, maturity, cellular circuitry, stromal barriers, and treatment context.
- Available ICC survival data establish the prognostic relevance of compartment-resolved TLS distribution, providing a foundation—but not yet direct validation—for multidimensional topotype assessment.
- Therapeutic development should aim to create candidate circuit-competent TLS niches and verify antigen presentation, lymphocyte renewal, and antitumor function, rather than simply increasing lymphoid aggregates.
- Pretreatment biopsy can characterize TLS states observed in sampled tissue, but whole-tumor topotype landscapes require confidence-graded integration of tissue coverage and complementary imaging information.

## 1 Introduction

ICC is a lethal primary liver cancer in which late diagnosis, marked intertumoral heterogeneity, desmoplastic stroma, and frequent postoperative recurrence limit the effectiveness of conventional and immune-based strategies (1–3). Immune checkpoint inhibitors and combination regimens have expanded the therapeutic landscape, but durable benefit remains restricted to selected patients, emphasizing the need for spatially informed biomarkers that explain why some tumors can be immunologically engaged whereas others remain cold or immune excluded (4–6).

TLS are ectopic lymphoid aggregates that can recapitulate selected features of lymph nodes, including organized T-cell and B-cell zones, germinal centers, dendritic-cell networks, and high endothelial venules (7, 8). In many cancers, mature intratumoral TLS correlate with improved survival and response to immune checkpoint blockade (9–11). This broad association has encouraged the use of TLS as a favorable biomarker, but ICC exposes a major limitation of that simple interpretation: TLS are not uniformly protective. Their prognostic and predictive meaning depends on where they form, which cells they contain, whether their architecture is functionally coupled to tumor antigen recognition, and whether they sustain checkpoint-sensitive antitumor immunity.

Rather than treating TLS as static histological structures or simply classifying them according to density and maturation, this review focuses on TLS as spatially programmed immune niches that shape tumor immune surveillance, restricted lymphocyte access, and checkpoint responsiveness in ICC. This perspective is particularly relevant to contemporary tumor immunology because TLS biology directly intersects with several central questions in cancer immunotherapy: how antitumor T-cell immunity is locally sustained, why immune infiltration does not always translate into clinical benefit, and how suppressive tumor microenvironments can be therapeutically reprogrammed.

We therefore propose “TLS topotype” as an operational, TLS-centered unit of spatial interpretation rather than an established pathological nomenclature. The primary unit is an individual TLS-centered region of interest that includes the TLS core, a prespecified surrounding neighborhood, and its relationship to the nearest tumor–stroma interface. At this level, topotype is a multidimensional state rather than a mutually exclusive category. It incorporates anatomic topology and tumor accessibility, structural maturity, putative effector circuitry, and suppressive context. Treatment-induced plasticity is assessed separately through longitudinal comparison of these domains. In ICC, this framework is particularly relevant because peritumoral fibrosis, antigen-presentation defects, myeloid dominance, and fibro-immune barriers may alter the biological meaning of otherwise similar lymphoid structures (12–14).

This differs from an immune archetype or spatial neighborhood, which may describe broader multicellular tissue states without requiring a TLS as the organizing unit, and from an ecotype, which generally captures recurrent ecosystem-level cell-state compositions rather than the functional topology of an individual lymphoid niche.

Three analytical levels should be distinguished. “TLS-level topotype” refers to the multidimensional state of an individual TLS-centered region. “Tumor-level topotype landscape” refers to the compartment-specific density and proportional distribution of multiple TLS-level states within a tumor. “Patient-level trial stratum” refers to a derived clinical grouping generated from the tumor-level landscape using a prespecified and validated algorithm. These terms are not interchangeable, and a patient should not be assigned to a single topotype solely according to one captured, predominant, or maximally suppressive TLS.

**Figure 1:**
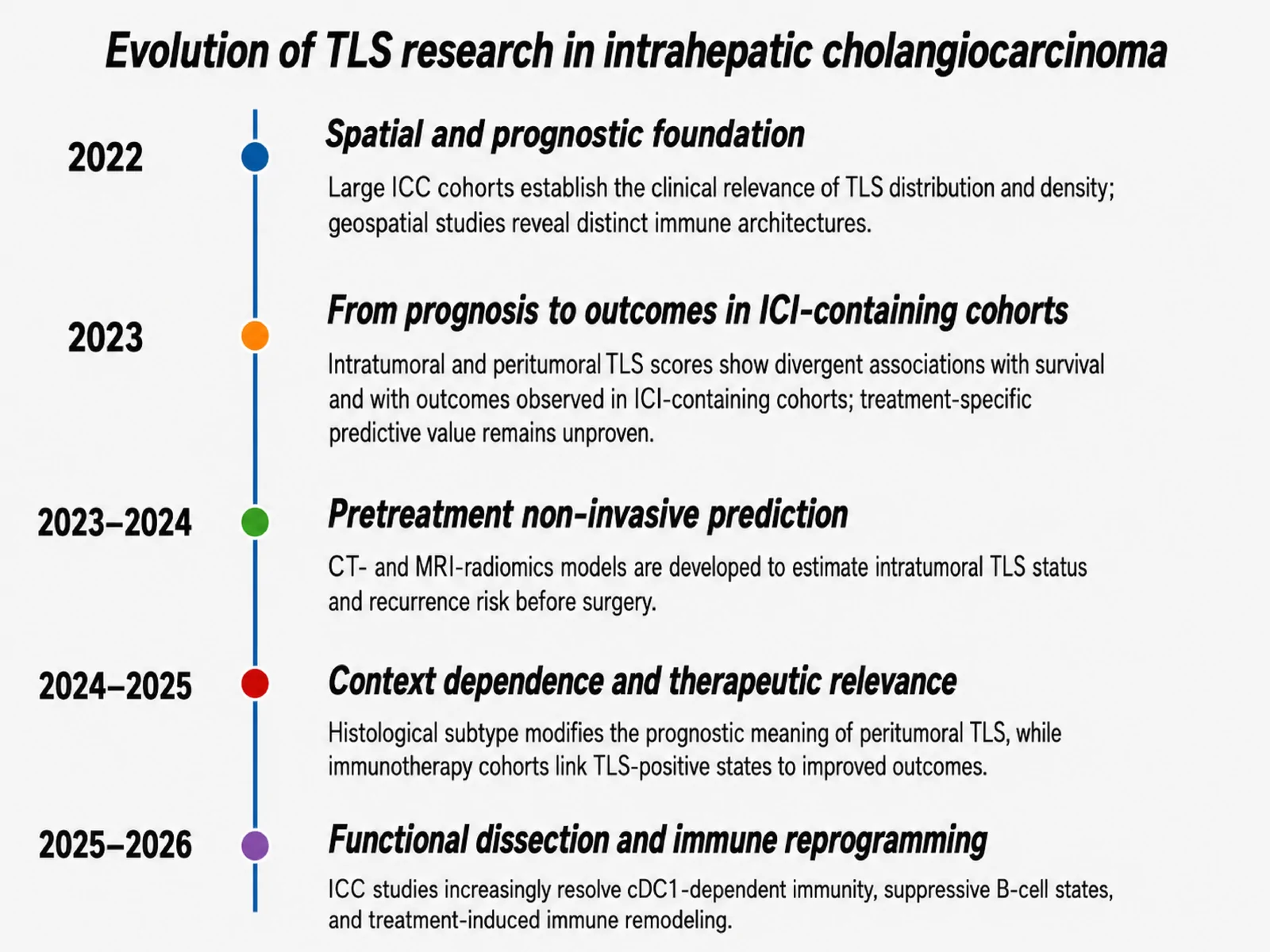
Evolution of TLS research in intrahepatic cholangiocarcinoma. The field has progressed from descriptive and prognostic studies of TLS spatial distribution toward immunotherapy-response stratification, non-invasive radiomic prediction, context-dependent interpretation across histological and molecular backgrounds, and mechanistic investigation of functional immune circuits. This timeline emphasizes that the proposed TLS topotype framework emerges from the convergence of these previously separate research directions rather than from a single classification system. TLS, tertiary lymphoid structure; ICC, intrahepatic cholangiocarcinoma; ICI, immune checkpoint inhibitor; CT, computed tomography; MRI, magnetic resonance imaging; cDC1, conventional type 1 dendritic cell.

The evolution of TLS research in ICC—from spatial description and prognostic association to treatment-response stratification, non-invasive prediction, and functional immune-niche modeling—is summarized in **Figure 1**.

## 2 Evidence-calibrated terminology

Throughout this review, we use “is associated with” for observational clinical findings; “may support,” “may contribute to,” or “is biologically compatible with” for mechanisms inferred from spatial, correlative, or cross-cancer data; and “candidate biomarker” when clinical utility remains unvalidated. The term “predicts benefit” is reserved for evidence demonstrating a treatment-by-biomarker interaction or an equivalent predictive analysis in an appropriately controlled dataset. Associations observed only within ICI-treated cohorts are therefore not considered sufficient to establish treatment-specific predictive value.

## 3 From TLS-level states to tumor-level topotype landscapes: an operational framework

The traditional TLS workflow asks three questions: is a TLS present, how mature is it, and how many are there? This approach is insufficient for ICC. A more translational workflow asks whether a TLS is anatomically positioned to interact with tumor cells, whether it contains candidate cellular configurations that may support antitumor immunity, and whether the surrounding stromal and vascular niche permits immune effector traffic. This shift transforms TLS from a histological descriptor into a testable immune organoid within the tumor ecosystem (15, 16).

Previous ICC and CCA studies have already evaluated TLS density, intratumoral versus peritumoral distribution, maturation, and selected immune-cell composition. Ding et al. combined intratumoral and peritumoral TLS scores to generate four geographic immune classes, whereas Shang et al. assessed compartment-specific TLS abundance, maturation, cellular features, and associations with immunotherapy outcomes (15–18). The proposed topotype framework is therefore not intended to rename or replace these established classifications. Its proposed incremental value lies in relational information not captured by counts or composition alone: whether cDC1, B-cell, and T-cell compartments form a spatially coordinated circuit connected to tumor nests; whether suppressive myeloid, Treg, or CAF neighborhoods interrupt this circuit; whether the associated stroma and vasculature permit tumor-directed immune traffic; and whether these relationships change during treatment (12, 19, 20).

The proposed framework may also help investigate the maturation paradox. Germinal centers, follicular dendritic cells, and high endothelial venules indicate structural organization, but structural maturity alone does not establish antitumor function. A mature-appearing TLS may remain disconnected from tumor-reactive circuitry or lie within a Treg-, macrophage-, or CAF-rich neighborhood. Conversely, a less mature intratumoral TLS may display candidate circuit-competent features if antigen-presenting, B-cell, and T-cell compartments are spatially coordinated and tumor accessible. These interpretations remain hypotheses to be tested rather than validated binary pathological classes (21–23).

At baseline, each evaluable TLS-centered region should be characterized across four cross-sectional domains: topology and tumor accessibility, structural maturity, putative effector circuitry, and suppressive context. Topology and accessibility include anatomic compartment, distance to tumor nests, tumor–TLS contact or interdigitation, and stromal or vascular barriers. Maturity includes T/B-cell organization, follicular dendritic-cell networks, germinal-center features, and high endothelial venules. Putative effector circuitry captures the spatial organization of antigen-presenting cells, B-cell help, and TCF7-positive CD8 T-cell populations. Suppressive context captures the density and proximity of Tregs, suppressive myeloid cells, macrophages, and CAF-associated barriers. These domains and their proposed measurements are summarized in **Table 1**. These four baseline domains should be measured independently and should not be collapsed into a single beneficial-versus-adverse axis. A TLS may simultaneously show high structural maturity, strong candidate effector circuitry, and a high suppressive burden. Conversely, an immature or only partly organized TLS may retain limited antigen-presenting or T-cell-supportive features. Accordingly, topology/accessibility, structural maturity, putative effector circuitry, and suppressive context should initially be reported as separate continuous or ordinal measurements. Mixed, discordant, and transitional profiles are expected and should be retained rather than forced into mutually exclusive categories.

**Table 1:** Operational multiscale framework for TLS-level topotypes and tumor-level topotype landscapes in ICC.

Domain	Unit/timing	Suggested measurements	Primary output	Aggregation/validation
Topology and accessibility	TLS-centered region; baseline	Compartment; tumor distance/contact; CAF barrier; vascular/HEV access	Continuous accessibility profile	Report by compartment; location alone is insufficient
Structural maturity	Individual TLS; baseline	T/B segregation; CD21/CD23 FDC network; BCL6/GC; PNAd/HEV	Ordinal or continuous maturity	Report maturity proportions; maturity is not function
Putative effector circuitry	TLS plus locked neighborhood; baseline	cDC1/mature DC; B–T–DC adjacency; Tfh/Tph; TCF7-positive CD8; chemokines	Candidate circuit profile	Report proportions/network metrics; colocalization is not functional proof
Suppressive context	TLS, neighborhood, and tumor interface; baseline	Tregs; MDSCs; TAMs/TANs; FAP/α-SMA CAFs; proximity metrics	Suppressive-neighborhood profile	Report burden/proximity; no “worst TLS” rule
Treatment-induced plasticity (Δ-topotype)	Paired samples; longitudinal only	Change in baseline domains and compartment-specific state distribution	Longitudinal change profile	Not a baseline additive axis; weights/cutoffs require external validation

Treatment-induced plasticity should not be treated as an equivalent baseline axis because it can only be measured using longitudinal or paired specimens. It is instead represented as Δ-topotype, describing treatment-related changes in the four baseline domains. We do not propose equal weighting or an unvalidated additive topotype score. Each domain should initially be reported separately; any composite weights and cutoffs must be derived in a training cohort, locked before validation, and tested for incremental value beyond TLS density, maturity, location, and immune-cell composition. Interobserver reproducibility, sampling requirements, and clinical utility also remain to be established.

Because multiple TLS states may coexist within one tumor, the primary tumor-level output should be a distributional topotype landscape. This report should include TLS density by compartment and the proportions of TLS showing candidate circuit-competent, suppressive-neighborhood, stromally excluded, or mixed/unclassified features. No single TLS, including the most suppressive structure, should automatically determine the whole-tumor designation, and a simple majority rule should not be assumed. If a single tumor- or patient-level stratum is required, it should be generated using a prespecified classifier that incorporates the full distribution and permits mixed or indeterminate assignments.

## 4 Topological context is a biological determinant, not a technical annotation

One of the most clinically relevant TLS variables in ICC is its anatomic compartment. Intratumoral TLS have repeatedly been associated with favorable immune phenotypes and improved outcomes in cholangiocarcinoma, whereas peritumoral TLS can correlate with worse survival or impaired immune penetration (17, 24). This distinction is not merely an artifact of sampling. It reflects whether lymphoid priming and B-T cell cooperation occur within the tumor antigen field or outside it behind stromal barriers.

**Figure 2:**
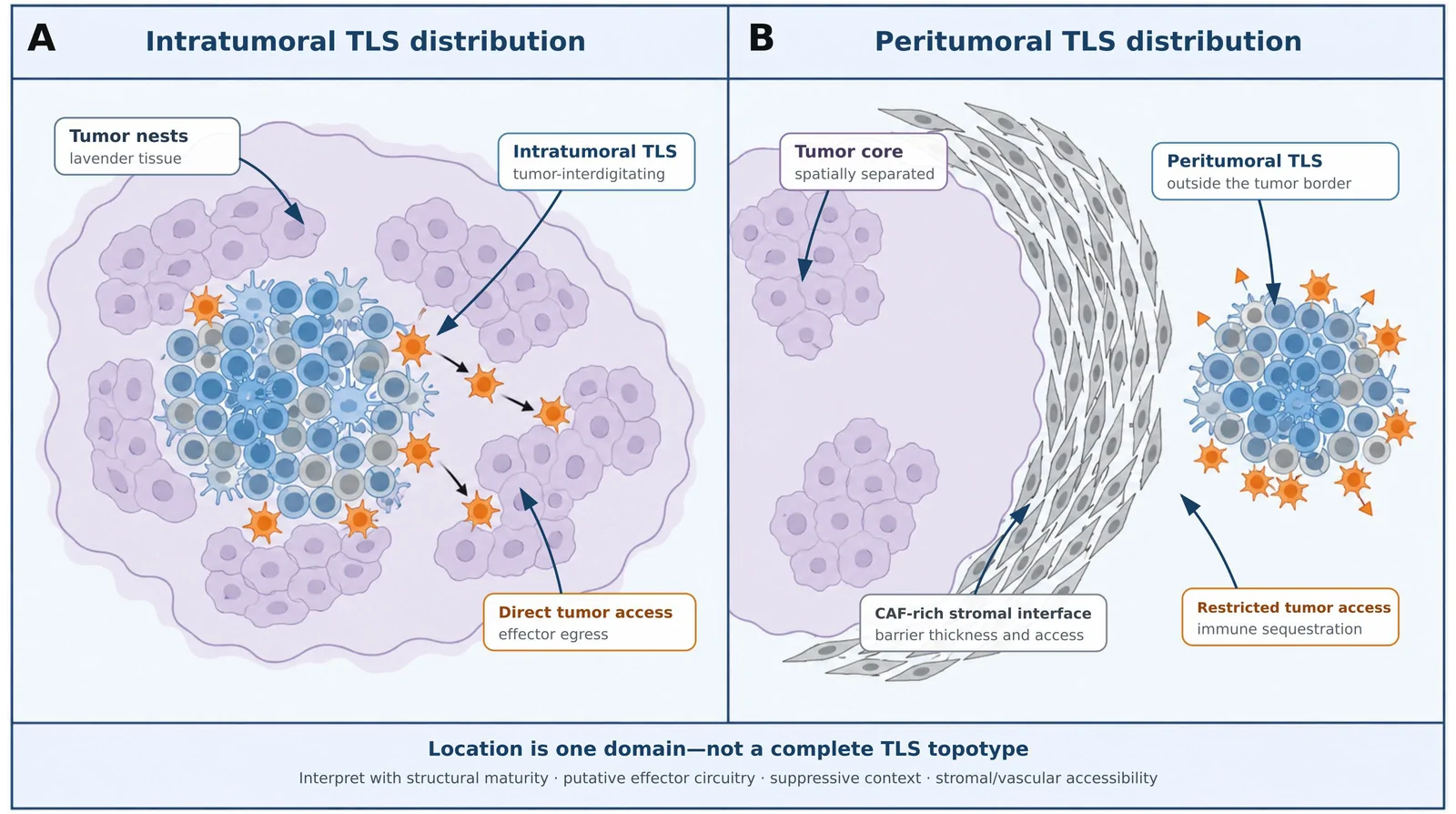
Anatomic compartment as one component of TLS topotyping in intrahepatic cholangiocarcinoma.Schematic representation of intratumoral and peritumoral TLS distributions in ICC. **(A)** Intratumoral TLS distribution. Intratumoral TLS are located within the tumor compartment and may be more directly connected to tumor nests, potentially facilitating effector-cell access to tumor tissue. **(B)** Peritumoral TLS distribution. Peritumoral TLS are located outside the tumor border and may be spatially separated from the tumor core by a CAF-rich stromal interface, potentially restricting direct immune access to tumor tissue. These patterns illustrate the established prognostic relevance of anatomic compartment but should not be interpreted as two complete or deterministic topotypes. Anatomic location alone does not define a TLS topotype; it must be evaluated together with structural maturity, candidate effector circuitry, suppressive context, and tumor accessibility. Intratumoral TLS may contain suppressive cells, whereas peritumoral TLS may support immune recruitment in selected contexts. Compartment should therefore be treated as a spatial variable rather than a surrogate for function.TLS, tertiary lymphoid structure; ICC, intrahepatic cholangiocarcinoma; CAF, cancer-associated fibroblast.

In ICC, TLS location should be interpreted as one component of functional context rather than as a complete topotype or a purely anatomical annotation. Intratumoral TLS are positioned within the tumor antigen field and may facilitate local interactions among antigen-presenting cells, B cells, and T cells, whereas peritumoral TLS may reflect stromal sequestration or, in selected contexts, an active immune niche. Accordingly, intratumoral, invasive-margin, and peritumoral compartments should be quantified separately, ideally with distance-based metrics, but location alone should not determine whether a TLS is considered candidate circuit-competent, excluded, or suppressive (17–20) (**Figure 2**). Importantly, intratumoral location does not imply an exclusively favorable immune state. In the large iCCA study by Ding et al., multiplex analysis showed that intratumoral TLS contained increased frequencies of both T follicular helper cells and regulatory T cells, while the frequency of Tregs within intratumoral TLS was associated with the peritumoral TLS score (18). These findings indicate that anatomical compartment and immune function are related but not equivalent. Suppressive immune components may coexist within an intratumoral TLS, and the intratumoral and peritumoral compartments may represent biologically connected rather than fully independent immune territories.

## 5 Prognostic associations of compartment-resolved TLS and the unresolved question of treatment-specific predictive value

Existing survival analyses establish the prognostic relevance of compartment-resolved TLS distribution, although they do not directly validate the proposed multidimensional topotype framework. In the largest dedicated iCCA study, Ding et al. evaluated 962 patients from three Chinese cancer centers and reported that the spatial distribution and density of TLS were significantly correlated with overall survival. Importantly, TLS located within and outside the tumor had different prognostic implications, and geographic integration of their distribution stratified iCCA into four immune subclasses with distinct clinical outcomes (18).

This topology-dependent prognostic pattern was independently reinforced by a CCA study including 471 surgically treated patients and 100 patients receiving first-line chemotherapy plus immune checkpoint inhibitors. A high intratumoral TLS score was associated with longer OS in both cohorts, whereas a high peritumoral TLS score was associated with shorter OS. In the surgical cohort, median OS was 29 months in the T-score-high group versus 17 months in the T-score-low group, while median OS was 14 months in the P-score-high group versus 22 months in the P-score-low group (17). Similar directional associations were observed in the immunotherapy-containing cohort, in which intratumoral TLS were associated with more favorable outcomes. However, because all patients in that cohort received an ICI-containing regimen and no appropriate non-ICI comparison group or treatment-by-biomarker interaction analysis was available, the study could not determine whether TLS specifically predicted benefit from checkpoint inhibition or represented a generally favorable prognostic immune context. Intratumoral TLS should therefore be regarded as a candidate biomarker for prospective evaluation rather than a validated predictor of ICI benefit. A subsequent single-arm phase II chemoimmunotherapy cohort similarly showed that TLS-positive tumors were associated with favorable response-related and survival outcomes and with inflamed B-cell- and T-cell-associated programs (24). However, the limited sample size and single-arm design precluded separation of treatment-specific predictive effects from general prognostic associations. These findings should therefore be interpreted as exploratory clinical associations supporting prospective biomarker development rather than as validation of an ICI-predictive TLS biomarker.

However, OS correlations are not uniform across all ICC contexts. Doi et al. examined 261 iCCA cases and found that high densities of both intratumoral and peritumoral TLS were associated with prolonged survival only in large-duct-type iCCA, with peritumoral TLS remaining prognostic in multivariable analysis for that histological subtype (25). This finding should not be regarded merely as an exception to a generally adverse interpretation of peritumoral TLS. Rather, the discordance between studies provides direct support for a context-dependent framework. Peritumoral TLS may reflect stromal sequestration and restricted tumor access in some tumors, but may be associated with active lymphocyte recruitment in vein-rich or large-duct-type microenvironments in others. Peritumoral location should therefore be interpreted together with histological subtype, vascular density, stromal organization, distance from tumor nests, and immune-cell composition rather than being used as a surrogate for a suppressive or non-functional TLS state.

Taken together, these studies establish compartment-resolved TLS density as a clinically meaningful prognostic layer in ICC and CCA. Associations observed in cohorts receiving ICI-containing therapy further support TLS as a candidate biomarker for prospective investigation, but they do not yet demonstrate treatment-specific predictive value. However, T-high/P-low, T-low/P-high, and related geographic categories should not be equated with the proposed TLS-level topotypes. Instead, they provide the established spatial foundation upon which measurements of candidate effector circuitry, suppressive context, stromal accessibility, and longitudinal change may add information. Future studies should evaluate the incremental prognostic and predictive value of tumor-level topotype landscapes separately. Prognostic analyses should report compartment-resolved Kaplan–Meier curves and multivariable Cox models for OS and should adjust for tumor stage, nodal status, resection margin, treatment, histological subtype, and established immune biomarkers. Treatment-specific predictive value should be evaluated in randomized or otherwise appropriately controlled cohorts using a prespecified treatment-by-biomarker interaction analysis rather than inferred from outcomes within an ICI-treated cohort alone.

## 6 Candidate circuit-competent TLS states: putative mechanisms that may support antitumor immunity

An important evidence boundary should be stated explicitly. Direct ICC data support spatial immune heterogeneity (12), B-cell dysfunction or immunosuppressive behavior (26), cDC1/dendritic-cell relevance (27, 28), and hyperactivated Treg states (29). By contrast, TLS-resident maintenance of TCF7-positive stem-like CD8 T cells, coordinated B-cell–dendritic-cell–T-cell circuits, and the causal link between such circuits and checkpoint responsiveness are derived largely from other solid tumors or non-ICC models (30–37). In this review, these mechanisms are therefore treated as biologically plausible hypotheses for ICC rather than established disease-specific facts; the current evidence boundary is summarized in **Supplementary Table 1**. To avoid conflating established ICC findings with cross-cancer extrapolation or emerging preclinical evidence, we apply a review-specific evidence tier in **Supplementary Table 1**: Tier 1, direct human ICC evidence with independent or multi-cohort support; Tier 2, direct human ICC evidence from a single cohort or disease-specific mechanistic study; Tier 3, human evidence from broader CCA or other solid tumors; and Tier 4, preclinical, cross-disease, or hypothesis-generating evidence. This hierarchy is descriptive rather than a formal GRADE assessment.

### 6.1 Stem-like CD8 T cells as a putative correlate of candidate circuit-competent TLS

A proposed translational value of TLS is not simply that they contain lymphocytes, but that functionally engaged TLS may support lymphocyte states associated with durable immunotherapy responses in other solid tumors. Recent single-cell and spatial studies, including preliminary iCCA conference-abstract data, suggest that functionally engaged lymphoid niches may support TCF7-positive stem-like CD8 T cells and related progenitor-exhausted T-cell states (30–33). This concept is crucial for ICC, where simple effector infiltration may be transient and insufficient in a suppressive hepatic microenvironment.

TCF7-positive CD8 T cells may provide a plausible mechanistic bridge between TLS and immune checkpoint blockade. Preliminary iCCA conference-abstract data localized TCF7-positive stem-like CD8 T cells to TLS and proposed an IgM–FcμR-mediated B-cell support mechanism (30), whereas most peer-reviewed mechanistic evidence still comes from melanoma, renal cell carcinoma, and other solid tumors (31, 32). Checkpoint inhibitors often depend on progenitor-like pools capable of expansion rather than solely reactivating terminally exhausted cells. A TLS-level state enriched for TCF7-positive CD8 T cells may therefore represent a candidate circuit-competent phenotype with potential prognostic or therapeutic relevance. However, the presence or colocalization of TCF7-positive cells does not by itself establish local renewal, tumor specificity, or functional responsiveness. Accordingly, a TLS-resident TCF7-positive progenitor pool in ICC should be regarded as preliminary evidence requiring independent, full peer-reviewed validation rather than as an established mechanism.

### 6.2 B cells and dendritic cells define whether TLS are immune organs or lymphoid shadows

B cells in TLS should not be treated as passive markers of maturity. Naive B cells, germinal-center B cells, plasma cells, and regulatory B cells have different functional implications. Within candidate circuit-competent lymphoid niches, B cells may support T-cell persistence, antigen presentation, antibody production, and local immune education. In ICC, restoration of B-cell functionality has been proposed as a means of enhancing antitumor immunity (26, 38). Recent human iCCA data also show that tumor-infiltrating B cells can acquire dysfunctional or immunosuppressive states (26), underscoring that B-cell abundance alone cannot define a functionally active antitumor TLS.

Dendritic cells constitute another proposed component of this circuit. cDC1-mediated cross-presentation is relevant to CD8 T-cell priming and ICC antitumor immunity (27, 28), while experimental evidence from non-ICC models indicates that dendritic cells can help maintain established TLS through lymphotoxin and homeostatic chemokines (34). These findings support assessing dendritic-cell content and spatial organization, but they do not yet prove a functional TLS-resident B-cell–DC–T-cell circuit in ICC.

**Figure 3:**
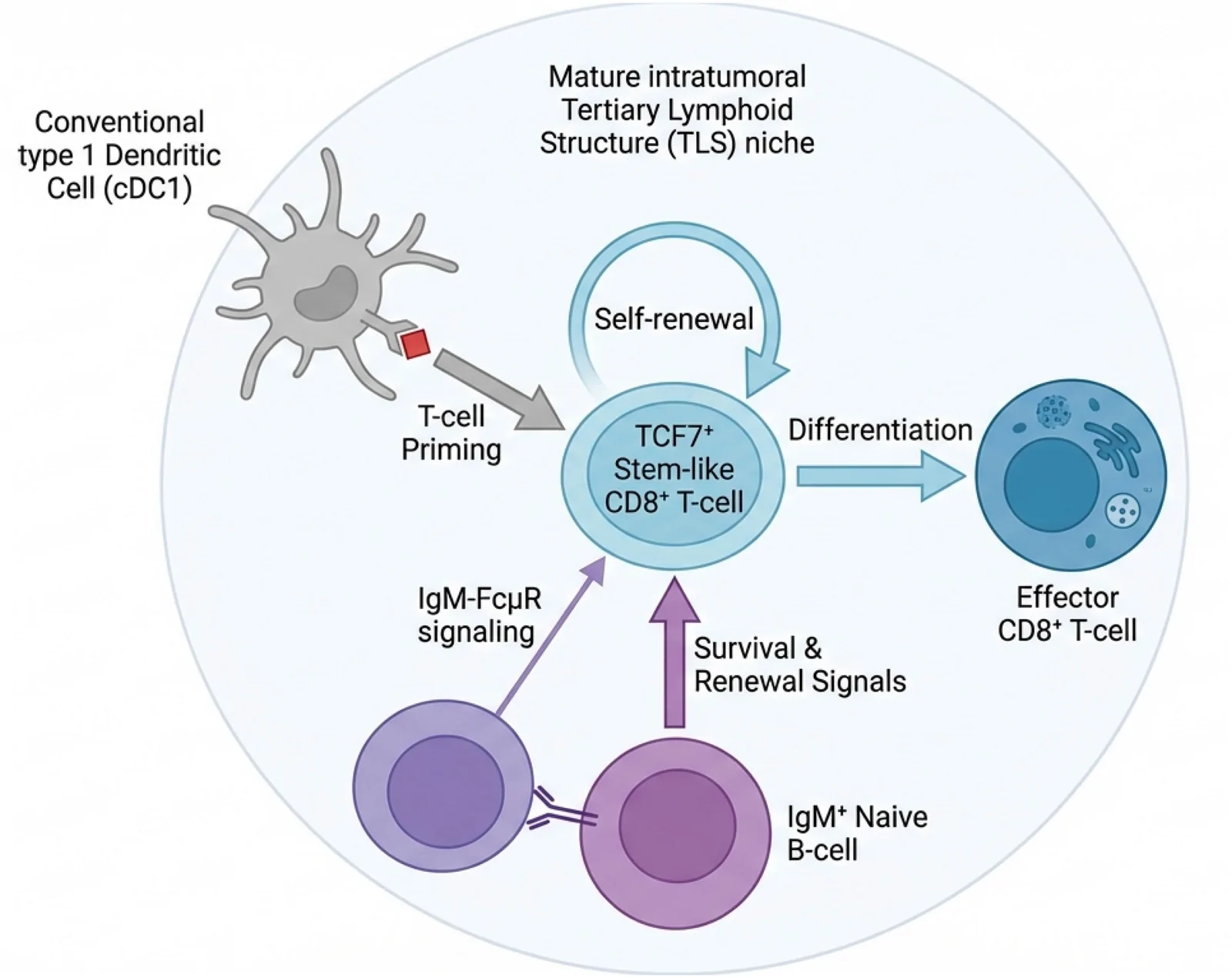
Hypothesized TLS-associated circuit involving stem-like CD8 T cells. Schematic illustration of a candidate cellular circuit that may sustain checkpoint-sensitive antitumor immunity within a tumor-connected TLS. cDC1-mediated antigen presentation, B-cell-associated signals, and TCF7-positive stem-like CD8 T cells are shown as putative interacting components. These spatial associations are hypothesis generating in ICC and do not alone demonstrate tumor-antigen specificity, local T-cell renewal, or checkpoint responsiveness. cDC1, conventional type 1 dendritic cell; TCF7, transcription factor 7; CD8, cluster of differentiation 8; IgM, immunoglobulin M; FcμR, Fc mu receptor; TLS, tertiary lymphoid structure.

These proposed circuits argue for a multidimensional spatial profile rather than a single marker. A candidate effector-circuit profile could include intratumoral or tumor-interdigitating location, organized B- and T-cell compartments, cDC1 or mature dendritic cells, Tfh/Tph-related signals, CXCL13/CCL19/CCL21 chemokines, and TCF7-positive CD8 T cells (35–37) (**Figure 3**). Marker expression and spatial colocalization support a candidate circuit-competent phenotype but do not by themselves demonstrate tumor-antigen presentation, tumor specificity, local T-cell renewal, or direct antitumor function. The term “functionally validated productive TLS” should be reserved for structures supported by such additional evidence.

## 7 Suppressive programs within and around TLS: coexistence, competition, and context dependence

Suppressive programs may coexist with, rather than replace, candidate circuit-competent or productive-feature-enriched TLS states. In ICC, Tregs, suppressive myeloid cells, dysfunctional B-cell states, macrophages, neutrophil programs, and CAF-associated barriers may coexist with organized T- and B-cell zones, follicular dendritic-cell networks, germinal-center features, or candidate effector circuitry within the same TLS-centered region. Productive-feature enrichment and suppressive burden should therefore be measured as independent and non-mutually exclusive dimensions. A TLS may score high on both dimensions, low on both, or show discordant values representing mixed, contested, or transitional states. The liver’s baseline tolerogenic immune environment further modifies the balance between these simultaneously present programs (29, 39–41). Tregs are a particularly important source of functional ambiguity. A TLS enriched with Tregs may preserve lymphoid architecture while suppressing the very T-cell responses it appears to support. This can explain why structural maturity does not always translate into favorable outcome. Biliary tract cancers also show heterogeneity in Treg and Th17-related immune states during progression, emphasizing that the cellular balance inside and around TLS should be considered a determinant of function (42).

Stromal suppression adds a second layer of ambiguity. CAFs can generate chemokines and extracellular matrix structures that organize immune cells, but they can also trap lymphocytes outside tumor nests and restrict access to the tumor core. In ICC, peritumoral myofibroblasts and fibro-immune interfaces may define the border at which an immune response is spatially detained (19, 20). Effective TLS function therefore requires not only immune competence but also stromal permeability.

**Figure 4:**
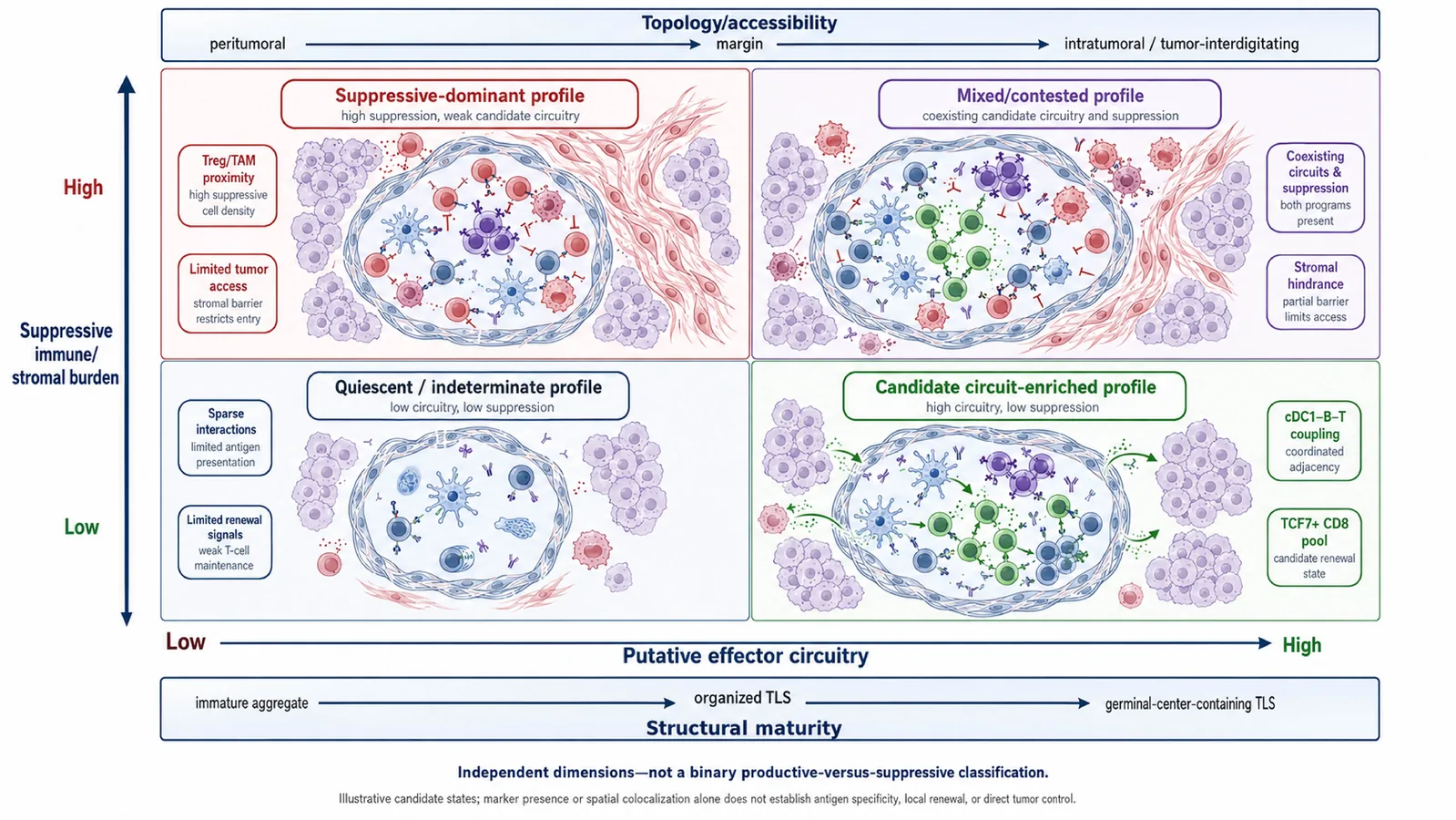
Multidimensional TLS states beyond a productive-versus-suppressive binary model. TLS-level states are represented by independent dimensions rather than mutually exclusive productive and suppressive categories. The horizontal axis represents putative effector circuitry, whereas the vertical axis represents suppressive immune and stromal burden. A TLS may show high values on both axes, low values on both, or discordant values corresponding to mixed, contested, suppressive-dominant, or indeterminate profiles. Topology/tumor accessibility and structural maturity are measured separately and do not determine placement within the matrix. Intratumoral TLS may contain suppressive components, whereas peritumoral TLS may support immune recruitment in selected histological or vascular contexts. Marker expression or spatial colocalization alone does not establish antigen specificity, local lymphocyte renewal, or direct tumor control. TLS, tertiary lymphoid structure; Treg, regulatory T cell; TAM, tumor-associated macrophage; cDC1, conventional type 1 dendritic cell; TCF7, transcription factor 7; CD8, cluster of differentiation 8.

Vascular regulation further shapes topotype. High endothelial venules can recruit lymphocytes and support TLS maturation, but endothelial programs can also become dysfunctional or disconnected from tumor-reactive immunity. Mechanistic studies demonstrate that endothelial Notch signaling can regulate TLS initiation and HEV-like vascular differentiation (43). For ICC, the key question is whether TLS-associated vasculature enables tumor-directed immunity or merely supplies a chronic inflammatory aggregate (**Figure 4**). These observations argue against a simple beneficial-versus-adverse TLS dichotomy. Mature-appearing structures may still contain Tregs or suppressive myeloid populations; human iCCA data indicate that B-cell states can be immunosuppressive (26); and peritumoral TLS have been associated with adverse outcome in some cohorts (17, 18) but favorable outcome in large-duct-type iCCA in another study (25). TLS effects may therefore vary with histological subtype, disease stage, treatment exposure, and molecular background. Accordingly, maturity and localization should be interpreted as context modifiers rather than deterministic labels.

### 7.1 Molecular background as a potential modifier of TLS topotypes

ICC genomic backgrounds are likely to modify the probability and function of TLS, but direct genotype-to-topotype mapping is currently lacking. In a CCA cohort, IDH1/2 mutations and FGFR2 alterations were associated with non-inflamed phenotypes and downregulation of antigen-presentation machinery (14). In contrast, a 226-patient ICC study associated FGFR2 fusion/rearrangement with lower Treg infiltration and a more immune-activated microenvironment (44), illustrating that the immunological effect of a driver alteration may depend on cohort, co-mutations, histology, and analytic method. ICC immune-subgroup analyses have also linked KRAS-mutant tumors to myeloid-inflammatory and MDSC-related programs (45), whereas TP53 alteration has been associated with resistance to ICI-based therapy in ICC (46).

At a broader level, the STIM classification identified reproducible inflamed and non-inflamed microenvironmental classes across approximately 900 iCCAs (47). These data support treating genotype as a potential modifier of TLS-level state and tumor-level landscape, not as a surrogate for either. At present, no evidence establishes that IDH1/2-mutant, FGFR2-fused, KRAS-mutant, or TP53-altered ICC consistently maps to a candidate circuit-competent or suppressive-neighborhood TLS state. Future studies should combine genomic profiling with compartment-resolved TLS pathology or spatial transcriptomics to test genotype–topotype interactions directly. The principal features, candidate biomarkers, and therapeutic implications of candidate circuit-competent versus suppressive-neighborhood TLS states are summarized in **Table 2**.

**Table 2:** Independent dimensions of TLS-level topotypes in ICC.

Dimension	Core measurements	Suggested output	Evidence status in ICC	Interpretation
Topology and tumor accessibility	Compartment, distance/contact with tumor nests, CAF barrier, vascular/HEV access	Compartment annotation plus continuous accessibility metrics	Tier 1: compartment-specific prognostic associations are supported, but direction varies by histological and vascular context	Intratumoral location is not inherently functional; peritumoral location is not inherently suppressive
Structural maturation	T/B-cell organization, CD21/CD23/BCL6, germinal-center and HEV features	Ordinal maturity score	Tier 2–3: structural features are measurable, but their functional meaning remains context dependent	Maturity does not prove antigen specificity, local renewal, or tumor control
Putative effector circuitry	cDC1/mature DC, B–DC–T spatial coupling, CD8/TCF7, Tfh/Tph and CXCL13-related signals	Continuous or relational candidate-effector profile	Tier 2 for cDC1 relevance; Tier 4 for a complete TLS-resident B–DC–TCF7^+^CD8 circuit	Indicates candidate immune activity, not validated antitumor function or ICI prediction
Suppressive immune/stromal burden	Tregs, suppressive myeloid/macrophage populations, dysfunctional B cells, CAF proximity and stromal barriers	Continuous suppressive-burden profile	Tier 2 for the ICC suppressive microenvironment; Tier 4 for direct TLS-centered functional mapping	May coexist with high maturity and strong candidate effector features
Treatment-induced change (Δ-topotype)	Paired changes in the four baseline dimensions	Dimension-specific longitudinal change	Tier 3–4: mainly exploratory, cross-cancer, or preclinical evidence	Increased TLS density alone does not establish favorable immune reprogramming

The four baseline dimensions should be reported independently rather than collapsed into binary productive-versus-suppressive categories. Mixed and transitional states should be retained, and marker colocalization alone does not establish function. At the tumor level, TLS-level measurements should be summarized as a distributional topotype landscape rather than reduced to the predominant or most suppressive TLS state.

## 8 Measuring TLS for clinical translation: multiscale assessment and biopsy-constrained interpretation

For TLS to guide treatment, measurement must be standardized, reproducible, and feasible across centers. The first tier remains routine pathology. H&E can identify lymphoid aggregates, their compartment, and their relationship to tumor nests. Immunohistochemistry or multiplex immunofluorescence can then validate T/B segregation, germinal-center markers, dendritic-cell content, and suppressive populations. The key is not to replace pathology with technology, but to use technology to formalize what pathology should report.

For routine pathology, a tiered rather than technology-maximal workflow is more realistic. A minimum first-line panel could combine H&E-based compartment annotation with CD3, CD20, and one follicular or maturity marker (CD21, CD23, or BCL6), reserving PNAd and candidate circuit or suppressive-context markers for selected cases. A second tier could add CD8, TCF7, cDC1/DC-LAMP, FoxP3, and FAP using targeted multiplex assays when the clinical or research question requires relational assessment; spatial transcriptomics should remain a reference-center validation tool rather than a routine requirement. Cross-institutional reproducibility will require prespecified compartment definitions, minimum evaluable tissue area, area-normalized density metrics, locked cutoffs, digital training sets, and external quality assurance (48, 49).

A translational TLS report in ICC should explicitly distinguish the level of inference. At the TLS level, it should include anatomic compartment, maturity, distance and contact with tumor nests, CD8 and TCF7-positive T-cell content, B-cell and plasma-cell composition, cDC1 or mature dendritic-cell markers, and Treg/myeloid/CAF proximity. At the tumor level, it should additionally report the number and area-normalized density of TLS by compartment and the proportional distribution of TLS-level states. The report should record whether assessment was performed on biopsy, resection, or post-treatment tissue, because these specimens support different spatial inferences.

### 8.1 Feasibility and limitations of biopsy-based TLS-state assessment before treatment

A critical practical issue is which TLS-level states can be observed in pretreatment biopsy specimens and how confidently these observations represent the whole-tumor topotype landscape. Tissue may be obtained by core needle biopsy or, in selected biliary tract cancer settings, by endoscopic ultrasound-guided tissue acquisition or transpapillary sampling rather than surgical resection (50). Biopsy-based assessment should therefore be regarded as constrained and confidence graded, not as a complete substitute for resection-based spatial mapping.

A biopsy specimen may positively identify a TLS-level state when a representative lymphoid aggregate and its relevant neighborhood are captured. Routine H&E/HES morphology with targeted immunohistochemistry, including CD20 and CD23, can assess TLS presence and maturation (15, 16, 48). Additional markers such as CD3/CD8, CD21/CD23 or BCL6, DC-LAMP or cDC1-associated markers, TCF7, FoxP3, myeloid markers, and CAF markers may support assessment of candidate effector circuitry and suppressive context (15, 16, 29–33). Such observations remain sample-level phenotypes unless broader spatial coverage is demonstrated.

However, a negative biopsy should not be interpreted as definitive TLS absence because TLS are spatially heterogeneous and may concentrate in intratumoral, invasive-margin, or peritumoral compartments. Surgical material has a higher likelihood of capturing TLS than biopsy specimens, illustrating the risk of sampling-related false negatives (17, 18). Biopsy reports should therefore specify the number of cores, total evaluable tissue area, tumor content, sampled compartment, completeness of the lymphoid aggregate, and confidence in the observed TLS-state assessment. When clinically safe and technically feasible, multicore or image-guided sampling should improve coverage of viable tumor and the tumor–stroma interface. A biopsy-confirmed TLS-level state should not be reported as a biopsy-confirmed whole-tumor topotype. Unless multiple spatial compartments are adequately sampled, the appropriate conclusion is “TLS state observed in the sampled tissue,” while the tumor-level landscape remains unresolved. Likewise, a biopsy without TLS should be reported as “TLS not detected in the sampled tissue” rather than “TLS absent.”

Digital pathology may further improve the reproducibility and scalability of TLS screening on available tissue sections. In a multicenter study of 1,924 patients with gastrointestinal cancers, an interpretable machine-learning model achieved accuracies exceeding 95% for detecting and classifying TLS into three maturation states on routine H&E-stained images (48).

### 8.2 Spatial multi-omics and computational refinement of TLS topotypes

Spatial transcriptomics and machine learning can extend this framework by assigning molecular states to visually defined TLS and their surrounding regions. Algorithms trained on spatial data can quantify neighborhood composition and model whether lymphoid structures are connected to candidate tumor-reactive immune circuits (51, 52). In ICC, these tools may help distinguish intratumoral TLS from peritumoral lymphoid aggregates and refine TLS-level states within broader immune archetypes (12). They remain tissue dependent and are primarily suited to mechanistic refinement and validation rather than routine pretreatment assignment of a complete tumor-level landscape from limited biopsy material.

### 8.3 Non-invasive and minimally invasive surrogates for pretreatment TLS assessment

Because biopsy may undersample spatially heterogeneous TLS, pretreatment assessment in ICC should not rely on one limited tissue sample alone (17, 18). CT- and MRI-based radiomics currently provide the most directly relevant non-invasive strategies. In ICC, preoperative contrast-enhanced CT radiomics and an MRI radiomics signature have been used to predict intratumoral TLS status and recurrence-free survival (53, 54). These studies support radiomics as a pretreatment enrichment tool for estimating whole-lesion probability or distribution of intratumoral TLS, but they do not measure a complete TLS-level topotype or establish a functionally validated tumor-level landscape.

Nevertheless, radiomics should be interpreted as a probabilistic whole-lesion surrogate rather than a replacement for pathology. Current ICC-specific models primarily estimate intratumoral TLS status and do not directly measure maturity, B–T-cell organization, dendritic-cell function, TCF7-positive CD8 reservoirs, suppressive cellular proximity, or stromal accessibility (53, 54). We therefore propose an integrated workflow in which biopsy-based pathology characterizes TLS states observed in sampled tissue, CT/MRI radiomics estimates whole-lesion TLS probability or distribution, and liquid biopsy provides complementary molecular context when tissue is limited. None of these modalities alone currently establishes a functionally validated tumor-level topotype landscape.

Liquid biopsy is particularly attractive in advanced BTC, where ctDNA-based profiling can complement tissue genomic analysis when adequate tumor material is difficult to obtain (55). However, current evidence supports ctDNA primarily for genomic profiling rather than direct inference of TLS architecture, relational circuitry, or suppressive spatial context. Circulating analytes should therefore be treated as exploratory molecular adjuncts, not direct TLS-level or tumor-level topotype classifiers. Future studies may test whether radiomics–liquid biopsy models improve prediction of resection-confirmed compartment-specific TLS distributions or outcomes, but such models require explicit tissue anchoring.

The strongest future models will likely integrate imaging, histology, spatial transcriptomics, circulating molecular features, and clinical variables. The minimum-to-advanced assessment layers for ICC biomarker studies are summarized in **Supplementary Table 2**.

### 8.4 Limitations and validation requirements of radiomics-based TLS prediction

Despite encouraging proof-of-concept results, radiomics-based TLS prediction remains vulnerable to several sources of bias. First, current ICC studies are predominantly retrospective and involve relatively modest cohorts, making performance estimates sensitive to case selection, event prevalence, and overfitting (53, 54). Second, radiomic features can vary across scanners, acquisition protocols, contrast phases, reconstruction parameters, segmentation strategies, and software pipelines, creating substantial risks of domain shift and limited inter-institutional reproducibility. Third, the pathological reference standard is itself imperfect: a resection-based TLS label may collapse heterogeneous intratumoral, invasive-margin, and peritumoral compartments into a single target, whereas biopsy-based labels may suffer from sampling error.

A further limitation is biological resolution. Current ICC-specific radiomic models primarily predict intratumoral TLS presence or probability rather than maturity, antigen-presenting competence, stem-like T-cell reservoirs, suppressive-cell proximity, stromal accessibility, or longitudinal change (53, 54). High discrimination for TLS status therefore does not constitute functional validation of a TLS-level topotype or tumor-level landscape. Before clinical implementation, models require protocol harmonization, locked external validation, calibration analysis, prospective testing, and benchmarking against compartment-resolved pathology; incremental value beyond established clinicopathological variables should also be demonstrated (53, 54, 56).

## 9 Therapeutic implications: build candidate circuit-competent TLS and verify function

### 9.1 Immunotherapy and chemoimmunotherapy as TLS-selective pressure

TLS are often discussed as biomarkers of immunotherapy response, but treatment may also reshape their spatial and cellular states. In solid tumors, mature TLS have been associated with immune checkpoint inhibitor efficacy independently of conventional PD-L1 assessment (11), and meta-analytic studies support associations between TLS and outcomes during immune checkpoint blockade (9, 10). In ICC, a baseline tumor-level topotype landscape should currently be regarded as a candidate stratification variable for prospective testing. Available CCA and ICC cohorts receiving ICI-containing therapy have shown associations between TLS features and clinical outcomes, but the absence of an appropriate non-ICI comparator in these studies prevents distinction between prognostic and treatment-predictive effects. Whether checkpoint blockade preferentially expands pre-existing candidate effector circuits in particular TLS landscapes remains to be tested using controlled treatment-by-biomarker interaction analyses.

However, ICC frequently lacks sufficient baseline immune activation. Combination strategies may be needed to convert a cold or immune-excluded tumor into one capable of forming tumor-accessible, candidate circuit-competent TLS states. Preclinical work suggests that chemotherapy and CTLA-4 blockade can reprogram the ICC immune microenvironment and enhance anti-PD-1 activity (57). Clinical chemoimmunotherapy and targeted-immunotherapy combinations provide the therapeutic setting in which tumor-level topotype landscapes and Δ-topotype should be prospectively tested (6, 58).

These observations motivate prospective evaluation of a response-adaptive strategy. A baseline tumor-level landscape may eventually support exploratory stratification into circuit-enriched and reprogramming-priority profiles, but such patient-level assignment requires a prespecified classifier and prospective validation. Early on-treatment biopsies or imaging could then assess whether therapy expands tumor-connected candidate effector circuits, reduces suppressive or stromal barriers, or merely causes nonspecific inflammation. This would convert the landscape from a static baseline profile into a pharmacodynamic readout.

### 9.2 Locoregional therapy and immunogenic injury

Locoregional therapies may be particularly relevant to ICC because they can release tumor antigens, remodel stroma, and induce inflammatory signals in a geographically restricted manner. Cryoablation combined with immune checkpoint blockade and lenvatinib has been investigated in advanced or metastatic ICC (59). From a TLS perspective, the critical question is not whether locoregional therapy increases immune infiltration in general, but whether it generates spatial changes compatible with antigen presentation, B–T-cell cooperation, and the maintenance or expansion of stem-like CD8 T-cell states. These outcomes should be measured directly rather than inferred from increased TLS density or marker colocalization.

This provides a rational design principle for trials: collect tissue before and after locoregional intervention, spatially map TLS-level states at the treatment margin and residual tumor core, quantify treatment-related changes in the four baseline domains as Δ-topotype, and correlate changes in the tumor-level landscape with response. Such studies would test whether local tumor injury converts peritumoral immune sequestration into tumor-accessible immune engagement.

### 9.3 Chemokine, stromal, vascular, and engineered lymphoid-niche approaches

Therapeutic induction of TLS has attracted substantial interest, but the goal should be functional lymphoid neogenesis. Chemokine and cytokine pathways such as CXCL13-CXCR5, CCL19/CCL21-CCR7, lymphotoxin-beta receptor signaling, IL-7, and Tfh/Tph-related IL-21 can potentially recruit and organize immune cells. Yet indiscriminate activation may worsen chronic inflammation or build suppressive TLS if the stromal environment remains exclusionary (35, 36, 60).

Stromal remodeling is therefore a central component of TLS-directed therapy. CAFs, extracellular matrix, and vascular programs determine whether lymphoid structures communicate with tumor nests or remain quarantined at the border. Strategies that normalize rather than ablate stroma may be needed, because stromal elements are also required to scaffold lymphoid organization (61). The therapeutic challenge is to convert a fibro-immune barrier into a permissive immune scaffold.

**Figure 5:**
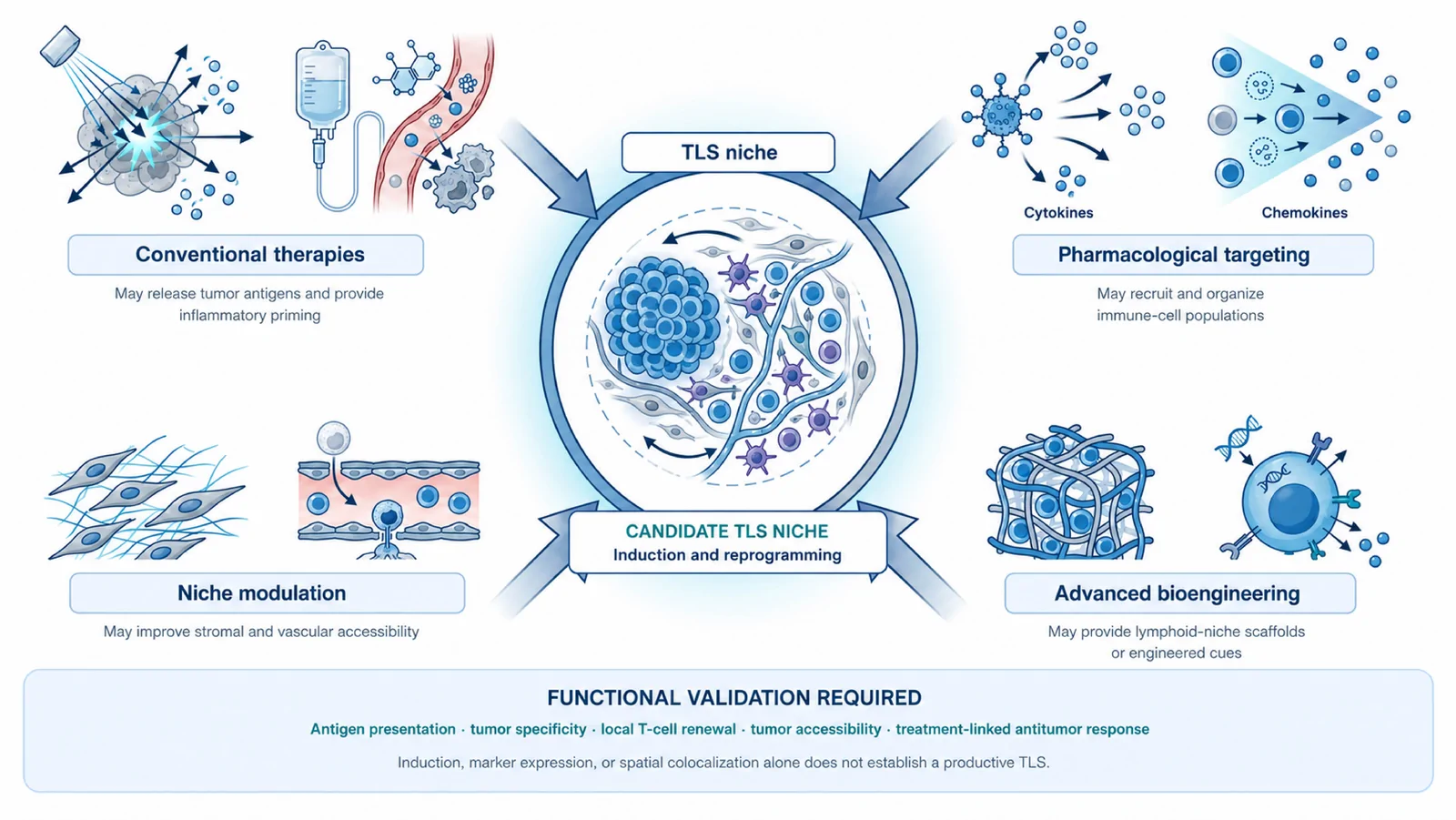
Therapeutic strategies for promoting candidate circuit-competent TLS states rather than merely increasing TLS density. Conceptual framework of treatments that may promote or reprogram candidate TLS circuits. Conventional therapies may increase antigen release and inflammatory priming; chemokine or cytokine modulation may recruit and organize immune cells; stromal and vascular remodeling may convert fibro-immune barriers into permissive scaffolds; and engineered platforms may provide lymphoid-niche cues. These strategies should be evaluated using functional endpoints—including antigen presentation, tumor specificity, cDC1 recruitment, TCF7-positive CD8 expansion, B-cell help, and tumor regression—rather than TLS number or marker colocalization alone. The proposed therapeutic relationships vary in evidence level and are predominantly hypothesis-generating in ICC; none currently establishes a validated treatment-selection biomarker.

More experimental approaches include epigenetic induction of TLS-related immune programs, STING activation, and biomaterial or mesenchymal stromal cell platforms designed to create artificial lymphoid niches (62). These approaches are conceptually attractive for ICC because they could be localized to the liver tumor microenvironment. Nevertheless, they should be evaluated with strict functional endpoints: TCF7-positive CD8 expansion, cDC1 recruitment, B-cell help, antigen specificity, and tumor regression, not only TLS number. The therapeutic logic of building functional rather than merely more TLS is illustrated in **Figure 5** and summarized across treatment modalities in **Supplementary Table 3**.

## 10 Proposed roadmap for TLS-driven translational trials in ICC

A TLS-driven trial should distinguish TLS-level measurement from patient-level stratification. The primary biomarker should be the tumor-level topotype landscape, including compartment-specific TLS density and the proportional distribution of candidate circuit-competent, suppressive-neighborhood, stromally excluded, and mixed/unclassified TLS states. For exploratory trial enrichment, these continuous and distributional variables may be summarized into candidate patient-level strata, such as circuit-enriched, exclusion-dominant, TLS-scarce, suppressive-neighborhood-dominant, and mixed/indeterminate profiles. These strata are not synonymous with individual TLS topotypes and should only be used after their assignment algorithm and cutoffs have been prespecified. Circuit-enriched profiles may be prospectively evaluated as enrichment variables in trials of ICI-containing therapy; Exclusion-dominant profiles may be evaluated in trials incorporating stromal or vascular remodeling; TLS-scarce profiles may be evaluated with antigen-release or lymphoid-neogenesis strategies; and suppressive-neighborhood-dominant profiles may be evaluated with myeloid- or Treg-directed combinations. Pretreatment evidence should be reported in confidence tiers: biopsy-observed TLS state, imaging-predicted whole-lesion TLS probability or distribution, integrated tumor-level profile, or indeterminate. Neither radiomics nor a limited biopsy should currently be described as establishing a functionally validated whole-tumor topotype. Prospective trials should predefine biopsy site, number of cores, evaluable area, imaging phase, segmentation method, and radiomic model. Whenever surgical tissue becomes available, sample-level and imaging-based estimates should be benchmarked against resection-based spatial pathology.

Several methodological safeguards are essential. Compartment definitions, tissue source, biopsy coverage, and evaluable area must be recorded because biopsy, resection, and post-treatment samples support different spatial inferences. TLS endpoints should be linked to pathological response, recurrence, progression-free survival, and overall survival. Compartment-resolved TLS density and tumor-level landscape variables should be prespecified for Kaplan–Meier and multivariable Cox analyses whenever follow-up is available, while any patient-level classifier must be locked before validation. Serial sampling or imaging should be incorporated where possible to distinguish prognostic, predictive, and pharmacodynamic effects.

Cutoff development is another priority. Intratumoral and peritumoral TLS density, maturity, candidate effector-circuit measurements, suppressive-neighborhood measurements, and accessibility should initially be retained as separate continuous and compartment-resolved variables. A tumor-level classifier may subsequently be developed using the full distribution of TLS-level states, but its weights and cutoffs must be trained, locked, and externally validated. Incremental value should be tested against nested reference models containing TLS density, maturity, intratumoral/peritumoral location, immune-cell composition, PD-L1, mismatch repair status, tumor molecular features, and relevant clinicopathological variables (46, 63).

A practical first validation program could use a prospective, multicenter two-stage design. Stage 1 would enroll untreated resectable ICC, obtain baseline core biopsies and standardized CT/MRI, apply a locked minimum TLS-state panel with central blinded pathology review, and compare biopsy-observed states and imaging estimates with resection-based tumor-level landscapes. Primary endpoints would include interobserver reproducibility, biopsy-to-resection concordance, and predefined sensitivity and negative predictive value. Stage 2 would embed a locked tumor-level classifier in an ICI-containing cohort, with ORR and 6-month PFS as prespecified biomarker endpoints and OS, pathological response, and Δ-topotype as secondary endpoints. A representative subset should undergo multiplex imaging and spatial transcriptomics. Predictive value should be tested using treatment–biomarker interaction, not inferred from prognosis alone.

**Figure 6:**
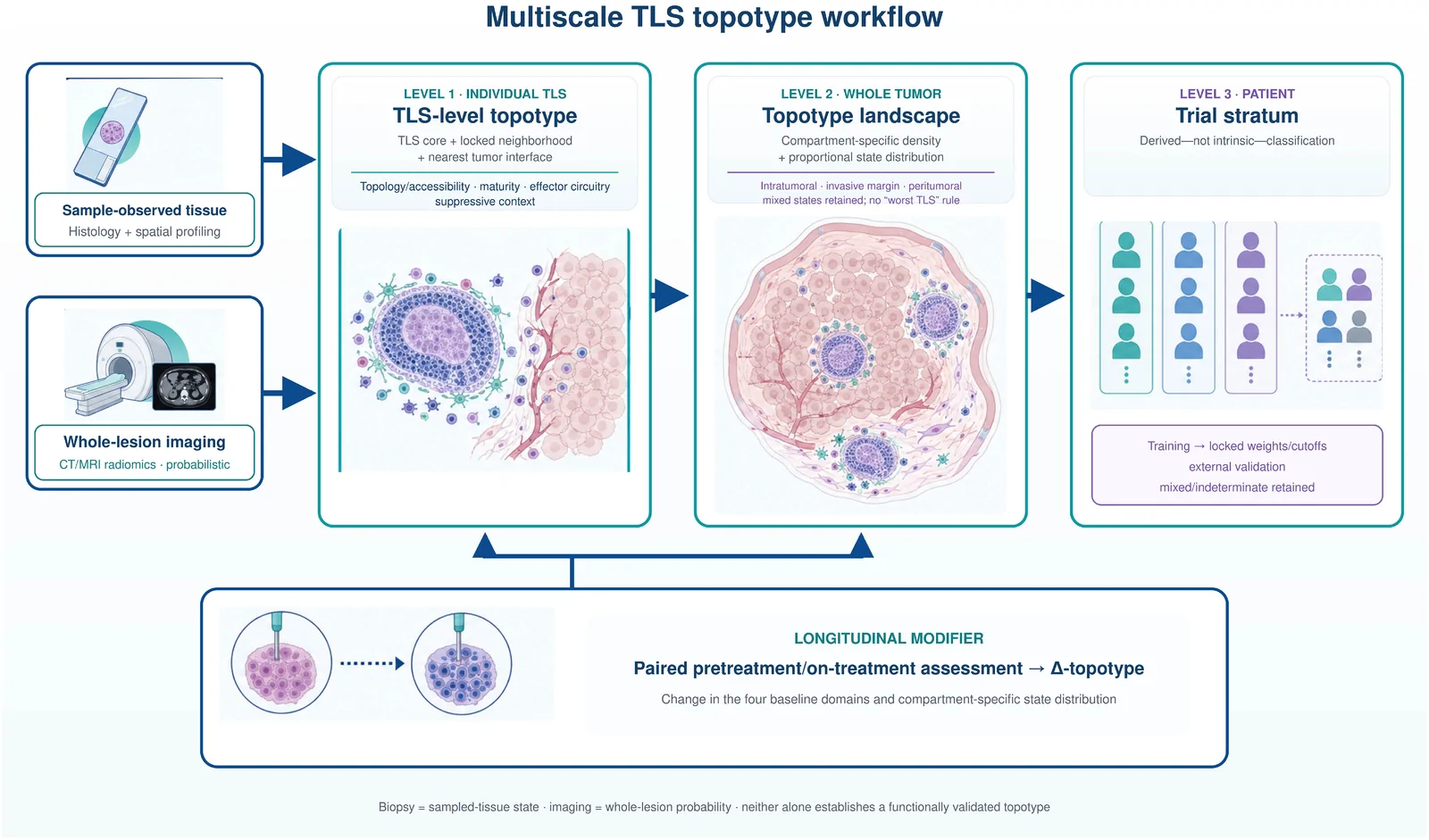
Multiscale workflow from TLS-level measurement to validated patient-level stratification. The individual TLS-centered region is the primary measurement unit. Four baseline domains—topology/accessibility, maturity, putative effector circuitry, and suppressive context—are recorded as a multidimensional TLS-level state. Compartment-specific densities and state proportions are then aggregated into a tumor-level topotype landscape. Only after prespecified training, locking, and external validation may this landscape be summarized as a patient-level trial stratum, with mixed or indeterminate assignments retained. Paired on-treatment assessment generates Δ-topotype as a longitudinal modifier. Biopsy provides sample-observed TLS states, imaging provides whole-lesion probabilistic information, and neither alone currently establishes a functionally validated tumor-level topotype. ICC, intrahepatic cholangiocarcinoma; TLS, tertiary lymphoid structure; CT, computed tomography; MRI, magnetic resonance imaging; Δ-topotype, treatment-induced change in the four baseline TLS topotype domains.

Finally, the field needs negative results. If a therapy increases TLS density without improving outcome, spatial and functional profiling should determine whether it produced an unfavorable or biologically inactive tumor-level landscape. Such studies would move the field away from assuming that more lymphoid tissue is always beneficial. The key translational endpoint should be verified immune function and tumor accessibility rather than lymphoid architecture alone (**Figure 6**).

## 11 Conclusion

TLS topotyping offers a testable framework for moving ICC biomarker development beyond TLS presence, density, maturation, and location alone. The individual TLS-centered region is the primary unit of biological interpretation, whereas the whole tumor is represented by a distributional topotype landscape. Patient-level trial strata are derived clinical outputs rather than intrinsic TLS topotypes. The framework’s proposed incremental contribution lies in determining whether a lymphoid niche is tumor accessible, spatially connected to candidate effector circuitry, dominated by suppressive stromal or myeloid programs, or altered by treatment.

Clinical translation will require standardized compartment and neighborhood definitions, reproducible minimum pathology panels, explicit sampling metadata, and prospective trials testing incremental value beyond TLS density, maturity, location, and cellular composition. Pretreatment biopsy should be interpreted as sample-level evidence and integrated cautiously with validated whole-lesion imaging estimates. Until the proposed measurements, aggregation rules, weights, cutoffs, and functional criteria are validated, TLS topotype should be regarded as an integrative hypothesis rather than a clinical pathological classification. Its value will depend on whether relational circuitry, suppressive spatial context, accessibility, or longitudinal change improves patient selection, guides rational combination therapy, or serves as a reproducible pharmacodynamic endpoint.

At present, no TLS-level state or tumor-level topotype landscape has been validated to predict differential benefit from immune checkpoint inhibition in ICC. Clinical use should therefore remain restricted to prospective biomarker-development and trial-stratification settings.
