# Supplementary material for: Tertiary lymphoid structure topotypes in intrahepatic cholangiocarcinoma: spatial immunity, checkpoint-response associations, and therapeutic reprogramming
Source: Front Immunol. 2026 Aug 20;17:1919543. doi: 10.3389/fimmu.2026.1919543 (PMC13539486; doi:10.3389/fimmu.2026.1919543)
Supplement: Supplementary file 1 [file Table1.docx]

**Supplementary Table S1. Evidence status of major TLS topotype mechanisms in ICC.**

| **Mechanistic proposition** | **Direct evidence available in ICC** | **Current evidence status** | **Evidence tier** | **Key validation gap** |
| --- | --- | --- | --- | --- |
| Spatial localization and prognosis | Large iCCA cohorts link intratumoral/peritumoral TLS distribution with OS, with context-dependent exceptions (17, 18, 25) | Strongest direct ICC evidence; topology is clinically relevant but not uniformly directional | Tier 1 | Prospective compartment definitions, locked cutoffs, and external validation |
| TCF7+ stem-like CD8 reservoirs within TLS | Preliminary iCCA conference-abstract evidence suggests TLS localization of TCF7+ stem-like CD8 T cells and B-cell support (30); peer-reviewed ICC confirmation is unavailable | Preliminary, hypothesis-generating direct ICC evidence, supplemented by cross-cancer data (31-33) | Provisional Tier 4 | Full peer-reviewed replication; spatial colocalization, clonality, self-renewal, and ICI-responsive expansion in ICC |
| B-cell function within TLS | Human iCCA data show dysfunctional/immunosuppressive tumor-infiltrating B-cell states (26) | Direct B-cell evidence, but not proof of a productive TLS B–T–DC circuit | Tier 2 | Subset-resolved spatial mapping, antigen specificity, and functional perturbation |
| DC/cDC1-dependent antitumor circuitry | ICC studies support cDC1/tumor-infiltrating DC relevance to antitumor immunity (27, 28) | Direct for DC biology; indirect for TLS-resident B-cell–DC cooperation | Tier 2 for ICC DC biology; Tier 4 for TLS-resident B–DC cooperation | Demonstrate TLS localization and causal interaction with B/T-cell compartments |
| TLS-mediated checkpoint responsiveness | No direct ICC-specific predictive evidence. Retrospective ICI-treated CCA cohorts and a small single-arm phase II chemoimmunotherapy cohort associated TLS with response-related and survival outcomes, but lacked non-ICI comparators and treatment-by-TLS interaction testing | Exploratory association evidence; prognostic and treatment-predictive effects cannot yet be separated | Tier 3 | Controlled ICC cohorts with prespecified treatment-by-TLS interaction analysis, calibration, and external validation |
| Suppressive TLS neighborhoods | iCCA data support hyperactivated Tregs and fibro-immune exclusion programs (19, 29) | Direct TME evidence; direct mapping of suppressive populations inside ICC TLS is incomplete | Tier 2 for ICC suppressive TME; Tier 4 for direct suppressive TLS mapping | Multiplex spatial proof within TLS and functional validation |
| Molecular subtype–topotype coupling | Immune/genomic subgroup data implicate IDH1/2, FGFR2, KRAS, and TP53 contexts (14, 44-47), but no direct genotype-to-topotype map exists | Hypothesis-generating | Tier 4 | Joint genomic profiling with compartment-resolved TLS pathology/spatial transcriptomics |

Evidence tiers are review-specific and indicate proximity to direct ICC validation rather than methodological quality alone. Tier 1 = replicated or multi-cohort human ICC evidence; Tier 2 = direct human ICC evidence from a single cohort or ICC-specific mechanistic study; Tier 3 = human evidence from broader CCA or other tumors; Tier 4 = preclinical, cross-disease, or hypothesis-generating evidence.

**Supplementary Table S2. Minimum and advanced TLS assessment layers for ICC biomarker studies.**

| **Layer** | **Core question** | **Feasible method** | **Added translational value** | **Major limitation** |
| --- | --- | --- | --- | --- |
| Routine morphology | Is there a lymphoid aggregate and where is it located? | H&E with explicit intratumoral/invasive-margin/peritumoral annotation | Low-cost screening and compartment assignment | Limited functional interpretation |
| Maturity phenotyping | Does the aggregate contain organized T/B zones and germinal-center features? | Minimum routine panel: CD3, CD20, plus CD21 or CD23/BCL6; PNAd optional | Distinguishes immature aggregates from organized TLS | Maturity may not equal antitumor function |
| Putative effector circuitry | Does an observed TLS show candidate antigen-presentation and stem-like T-cell features? | Second-tier targeted multiplex IF/IHC for CD8, TCF7, cDC1/DC-LAMP, FoxP3, FAP | Links sample-observed TLS states to ICI-relevant biology | Requires standardized panels and spatial analysis |
| Suppressive neighborhood | Is TLS hijacked by Tregs, myeloid cells, or CAFs? | FoxP3, CTLA-4, CD68/CD163, SPP1, FAP/alpha-SMA, spatial proximity analysis | Identifies suppressive-neighborhood features and combination needs | Cutoffs remain unvalidated |
| Non-invasive prediction | Can imaging estimate whole-lesion TLS probability or distribution before treatment? | MRI/CT radiomics, deep learning, integrated clinical models | Supports enrichment and serial monitoring; does not establish functional topotype | Requires multicenter validation and tissue anchoring |

**Supplementary Table S3. Therapeutic strategies viewed through the TLS topotype framework.**

| **Strategy** | **Expected TLS effect** | **Best-fit clinical scenario** | **Combination logic** | **Safety/translation concern** |
| --- | --- | --- | --- | --- |
| ICI alone | May expand pre-existing candidate TLS circuits | Circuit-enriched tumor-level profile | Anti-PD-1/PD-L1 with biomarker monitoring | Limited activity in cold or excluded tumors |
| Chemoimmunotherapy | Antigen release plus microenvironment reprogramming | Immune-low but reprogrammable ICC | Gemcitabine/cisplatin-based therapy with PD-1/PD-L1 or CTLA-4 blockade | Need to distinguish immune activation from nonspecific inflammation |
| Locoregional therapy | Spatially focused antigen release and inflammatory remodeling | Localized or oligometastatic lesions suitable for ablation or regional therapy | Ablation plus ICI/anti-angiogenic therapy | Risk of peritumoral aggregates without intratumoral penetration |
| Stromal/vascular remodeling | May convert fibro-immune barriers into permissive scaffolds | Exclusion-dominant profile with peritumoral TLS and immune-excluded core | CAF, matrix, or vascular normalization plus ICI | Over-suppression of stroma may impair TLS scaffolding |
| Chemokine/cytokine engineering | May promote recruitment and organization of B cells, T cells, and DCs | TLS-poor tumors with adequate antigenicity | CXCL13/CCL19/CCL21/LT-related strategies plus checkpoint blockade | May promote suppressive or autoimmune-like lymphoid structures |
| Artificial TLS platforms | Potential de novo construction of local immune niches | Highly resistant, immune-desert tumors in trial settings | Biomaterial or cell-based niche plus antigen and checkpoint strategy | Delivery, durability, and safety require careful validation |
